# The complexity of the relationship between neuropsychological deficits and impairment in everyday tasks after stroke

**DOI:** 10.1002/brb3.371

**Published:** 2015-09-16

**Authors:** Marta M. N. Bieńkiewicz, Marie‐Luise Brandi, Charmayne Hughes, Anna Voitl, Joachim Hermsdörfer

**Affiliations:** ^1^ Department of Sport and Health SciencesInstitute of Human Movement ScienceTechnische Universität MünchenMünchenGermany; ^2^ Graduate School of Systemic NeurosciencesLudwig‐Maximilians‐Universität MünchenPlanegg‐MartinsriedGermany

**Keywords:** Action disorganization syndrome, activities of daily living, apraxia, neuropsychological deficits, stroke

## Abstract

**Background and purpose:**

A large body of research reports that stroke patients are debilitated in terms of daily independence after dismissal from the hospital unit. Patients struggle with the use of daily objects or performing complex actions. Differences between individual deficits of patients are often associated with the site of the brain damage. However, clinical studies suggest that patients exhibit varied constellations of action‐associated difficulties and neuropsychological deficits. There is a lack of conclusive evidence indicating how different neuropsychological symptoms link to the impaired ability to perform activities of daily living (ADL).

**Materials and methods:**

To further address this matter, in this study we compared the behavior of patients with left brain damage (LBD) and right brain damage (RBD) following stroke in two naturalistic task scenarios (tea making and document filing), and compared the committed action errors to the neuropsychological screening results.

**Results:**

We observed mild to severe impairments in both the LBD and RBD groups amounting to 37–55% of failure rate in attainment of action goal. Interestingly, the performance on both tasks was not correlated to each other, suggesting that the tasks involved a different set of higher cognitive functions. Despite similar behavioral manifestations, in the LBD group poor task performance was related to deficits in praxis performance and unilateral tactile and visual extinction. The presence of aphasia did not correlate with task performance, except for a link between low scores in Aachen aphasia test scales and misestimation error in the tea making task. In the RBD group, difficulties with performance were primarily linked to deficit in praxis and unilateral visual extinction.

**Conclusions:**

Despite similar behavior, the underlying mechanisms of the deficits after stroke might be different (in patients with LBD and RBD) and reveal complex interlinks of cognitive networks involved in the ability to carry on everyday tasks.

## Introduction

Apraxia and action disorganization syndrome (AADS) affects a significant amount of patients in the postacute phase of stroke. According to the widely accepted definition, apraxia (Rothi and Heilman 1997) is treated as impairment in tool use, gesturing, and imitation that is independent from sensory and motor impairments of stroke. Usually apraxic behavior is associated with left brain damage (LBD) following a stroke and varies in clinical manifestations. Action disorganization syndrome is by some researchers differentiated from apraxia as a resultant of right brain damage (RBD) or frontal lobe damage (Humphreys and Forde 1998), and in the simplest description means impairment of the ability to organize multistep actions in a chain of subtasks. For the purpose of this article, we will not disambiguate those two syndromes, but assume that AADS is an umbrella term for behavioral manifestations of the inability to perform tool‐oriented, meaningless, and communicative gestures. Patients can suffer from difficulties with actual object use as well as the use of nonverbal communication and pantomime (Goldenberg 2013). Pantomime performance (i.e., demonstrating tool use without holding the object) is regarded as one of the core features of apraxic difficulties (among impaired tool use and imitation of meaningless gestures). Although pantomime does not include every feature of the movement sequence that would occur during the actual manipulation of the object, it is believed to reflect the core features of “simulated” use (Buxbaum et al. 2014). According to cognitive conceptualizations of apraxia, if the access to, or the concept of use, is impaired then patients will not be able to demonstrate how they would use an object. Hermsdörfer et al. (2012) used a “scooping” motion task, and demonstrated that pantomime performance may translate into the actual tool use. However, the relationship between the actual tool use, performance in naturalistic scenarios, and pantomime can vary between patients following stroke (Buxbaum et al. 2000; Bickerton et al. 2012). As proposed by Bienkiewicz et al. (2014), we grouped difficulties with motor performance in naturalistic task scenarios into three categories of deficits—sequencing, concepts of use including gesture knowledge, and spatiotemporal features. This taxonomy partially overlaps with the categorization of apraxic pantomime and imitation errors proposed by Hoeren et al. (2014), namely content and movement errors. In comparison, Hartmann et al. (2005) did not use an error classification in their study, but a percentage scoring for the naturalistic task performance. In the paper of Buxbaum (1998) overall error scoring was used with ratio of error types. Many authors distinguish more error categories that provide more detailed description of the AADS difficulties (for review see Goldenberg 2013, Chapter 9).

Despite the plethora of research describing the different difficulties that patients have when performing activities of daily living (ADL)‐like tasks (Buxbaum 1998; Humphreys and Forde 1998; Schwartz et al. 1999; Sunderland et al. 2006; Bickerton et al. 2007, 2012), there is no clarity regarding the link between the deficits that patients exhibit and other neuropsychological syndromes that can occur as a consequence of stroke. According to Katz et al. (1999), around 30% of ischemic stroke survivors suffer from comorbid to motor impairment signs of stroke. Depending on the hemisphere and the location within the damaged hemisphere, the patient can suffer from motor disability, impairment of language production and comprehension (aphasia), cognitive decline, spatial attention difficulties (neglect), and visual deficits (hemianopia). There is conflicting evidence about the links between those syndromes and the ability to perform ADL‐oriented tasks. For example, in patients with RBD it was demonstrated that visuospatial deficits are related to the outcome of rehabilitation in the posthospitalization phase (Denes et al. 1982; Jehkonen et al. 2006; Walker et al. 2012). Wade and Hewer (1987) added that hemianopia is a secondary important predictor for the functional outcome. Jehkonen et al. (2000) argued that other symptoms such as hemiparesis and language deficits were reported to lack predictive power. In the study of Hartmann et al. (2005), performance of aphasic LBD patients in multistep actions correlated with the severity of aphasia for preparing coffee, but not for fixing and starting a tape recorder. In the same tasks, patients with RBD demonstrated difficulty with keeping track of the multistep action performance and their performance correlated with the severity of hemineglect. Likewise, Schwartz et al. (1999) reported that RBD patients had a variety of action errors correlated with the severity of their hemineglect, which was not limited to the left side of the work space. Consistent with the presented evidence, Katz et al. (1999) argued that rehabilitation strategies for stroke patients should be oriented on the individual neuropsychological symptoms in order to increase independence of daily living. However, the extent to which the neuropsychological syndromes comorbid to AADS play a role in limiting daily functioning remains a subject of debate.

In this study, we investigated the relationship between aphasia, spatial attention (neglect and extinction), and other neuropsychological symptoms occurring as a consequence of stroke in the context of ADL performance. On the basis of previous reports, we expected to find similar extent of ADL deficits in both groups of patients. We hypothesized AADS impairment can be linked to the severity of other perceptual and cognitive deficits observed in both LBD and RBD stroke survivors. Precisely, we expected that difficulties with ADL‐like tasks correlate with not only gestural praxis but also with aphasia in LBD sample, and with spatial attention in RBD patients.

## Methods

### Participants

In all, 38 LBD patients were included in the analysis along with 17 RBD patients. The mean age for the LBD patient group was 58 years (SD = 12 years) and for the RBD patient group was 61 years (SD = 12 years). The LBD group comprised 20 males and 18 females and the RBD group comprised nine males and eight females. All participants suffered from a first stroke and were tested within the range of 2 weeks poststroke up to 4 months. All participants were patients of the ward of the Department of Neuropsychology at the Hospital Bogenhausen Munich or attended the outpatients’ clinic. Recruited patients suffered from typical neuropsychological syndromes following stroke, such as aphasia, apraxia, neglect, other spatial deficits, and deficits of attention. Patients with history of previous stroke, nonstroke‐related neurological problems, psychiatric disorders, substance abuse history, or an inability to understand instructions were excluded from recruitment.

We tested 12 age‐matched control subjects in the tea making (TM) task. All were neurologically healthy (*M* = 58.1, SD = 13.4 years). Six females and six males were recruited (10 right‐handed, 2 left‐handed, all performed the task bimanually). In the document filing (DF) task, we tested 12 age‐matched control subjects (*M* = 55.8, SD = 15.1 years), including six females, all participants were right‐handed and performed the task bimanually. Table 1 shows demographic and clinical data of the participants. No significant difference was found in terms of age between the control and patient groups with one‐way analysis of variance, *F*(3, 75) = 0.45, *P *>* *0.05.

**Table 1:** Demographic and clinical data

Patient group	LBD	RBD
	*n* = 38	*n* = 17
Gender: female/male	16/22	8/9
Age	58.9 (34–89)	61 (44–85)
Days since stroke	99.4 (18–269)	127.3 (27–281)
Education: Vocational/Middle School/Academic	8/16/14	5/9/3
Aetiology: Ischemia/Bleeding/Both	26/11/1	12/4/1
Locus: MCA/ACA/PCA/ICB/MCA plus/CB/TH/BG/NA	17/2/–/6/5/3/–/1/4	3/1/4/–/2/2/1/–/4
Patients with neglect	–	14
Patients with hemianopia	7	5
Hemiparesis/plegia	20	12
Aphasia: No, Amnesic, Anomia, Broca, Global, Non‐classificable, TMA, Wernicke	8/5/1/7/8/3/6	–

Values in brackets denote range of values.

Cortical: MCA, middle cerebral artery; ACA, anterior cerebral artery; PCA, posterior cerebral artery; ICB, intra cerebral haemorrhage; MCA plus, MCA with subarachnoid bleeding or MCA with PCA. Subcortical: CB, cerebellar infarction; TH, thalamus infarction; BG, basal ganglia; NA, unknown; TMA, transcortical motor aphasia.

### Ethical considerations

This report is based on the clinical screening for the CogWatch project conducted in the Hospital Bogenhausen Munich. The study design was approved by the ethical committee of the Medical Faculty of the Technical University of Munich. Informed consent was obtained from all subjects, and the study was conducted in accordance with the Declaration of Helsinki. Participation was voluntary, and patients were informed that they could withdraw at any time without giving a specific reason and without their treatment at the hospital being affected.

### Procedure

The testing procedure comprised scales of a Birmingham Cognitive Screen (BCoS) (Humphreys et al. 2012): praxis, spatial, and controlled attention sections; and two naturalistic tasks: tea making and document filing. BCoS sections included were as follows: complex figure copy multistep object use (MOT), gesture production, gesture recognition, gesture imitation (praxis subscales), apple test (used for the evaluation of spatial attention), and visual and tactile extinction scales. The BCoS assessment approximately takes 30 min and allows for the testing of aphasic individuals, as the patients are allowed to respond nonverbally. The complex figure copy task, similar to the Rey–Osterrieth figure copy test (Meyers and Meyers 1995), assesses participants’ ability to copy a complex, but meaningless rectangular shape comprised many geometrical features. The recent study by Chechlacz et al. (2014) showed that performance on this task involves a number of complex cognitive processes such as spatial coding, attention, motor execution and planning, and a discrete network of neural substrates.

The MOT assesses patients’ ability to select adequate components (among distractor items) and assemble (with the batteries placed in the correct orientation) and switching on a torch. This scale aims to provide an overview of performance capacity in everyday like context, and performance in this task is reportedly independent from the performance on praxis scales (Bickerton et al. 2012; Humphreys et al. 2012). Praxis subscales cover cognitive processes that support praxis such as action semantics, coding of body parts, input processing of visual stimuli, and gesture output processing. Gesture production assesses the ability to produce pantomime to auditory command from the examiner. In gesture recognition tests patients were asked to recognize communicative gestures and pantomime demonstrated by the examiner (aphasic patients are given forced choice options), and in gesture imitation patients are ask to imitate a meaningless gestures. The praxis subtests comprised three to six items each. The apple cancelation test is designed to measure allocentric and egocentric types of neglect (Chechlacz et al. 2012, Bickerton et al. 2011). Driver and Vuilleumier (2001) conceptualized neglect as a bias to favor stimulus on the ipsilateral side and dismiss the stimuli presented in the contralesional peripersonal space after unilateral brain damage. Participants are presented with a printout in a landscape orientation with 50 whole apples spaced out geometrically among other distractors (i.e., apples with an opening on either the left or right side). Egocentric neglect is assessed on the basis of the tendency to miss apple targets in the section of the sheet. Allocentric neglect is measured as number of false positive responses (canceled apples with opening on the right or left side). In addition, visual and tactile extinction were included in the assessment to assess difficulty with attending to two competitive stimuli at the same time as an aspect of neglect syndrome. The extinction phenomenon is particularly detrimental when multiple stimuli compete at once for attention, particularly in ADL context (Driver and Vuilleumier 2001). In the visual extinction task the examiner sat across from the patient with the arms lifted on either side of the head (1 m away with the hands approximately 20 cm from the nose). The examiner moved the index finger on the right/left hand or bilaterally for a brief moment, and the participant was asked to fixate on examiners nose and point out which finger/s was bent. In the tactile part of this assessment, the participant was asked to keep the eyes closed and sit straight on the chair. The examiner used an identical protocol to the visual extinction test and tapped twice on the dorsal surface of the patient's thigh (left/right/bilaterally). For visual and tactile extinction scales, the extinction index was calculated as difference between the right and left extinction score, and quantified as a difference between the bimanual and unilateral performance (Chechlacz et al. 2013).

The experimental procedure took an hour to administer, but had to be completed within two sessions for some patients. In addition, the Aachen aphasia test (Huber et al. 1984) was administered to LBD patients in order to assess their level of language comprehension. This test comprises assessment of spontaneous speech and comprehension, retention, and written language, and was conducted by a speech therapist of the Hospital Bogenhausen Munich in a separate session. For analysis, token test (TT), naming, comprehension, repetition, and written language percent rank (PR) scores were included. In addition, presence of hemiparesis, hemianopia, and neglect was retrieved from the medical records in the hospital. In this study we used two error taxonomies to describe the behavior of patients. First one consisting of more detailed taxonomy system that classifies types of action errors (Table 2). Further, error types were grouped into three global categories; sequencing errors, conceptual errors, and spatio‐temporal errors (Bienkiewicz et al. 2014) in order to examine correlations between the neuropsychological syndromes and the difficulties exhibited by patients. Sequencing errors included action: addition, anticipation, omission, perplexity and perseveration. Some of the error categories originally classified as ingredient omission or ingredient substitution were classified as conceptual errors. In addition, the conceptual error category comprised of substitution, misestimation, and quality errors. Spatio‐temporal category incorporated execution, toying, misuse, and mislocation errors.

#### TM task

Participants were comfortably seated in front of a table with a dimension of 180 × 80 × 70 cm (L × W × H). All of the objects were located in the reachable distance of participant (for detailed descriptions see Hughes et al. 2014, who used the same paradigm). Each participant was verbally asked to make two cups of tea, one with milk and two sweeteners, and another one with lemon and one sugar cube. Subjects were informed that all the things required to make the tea are on the table, and no time constraints were placed on task performance. A picture showing ready cups of tea with the ingredients listed was displayed by experimenter in front of the patient at the height of eyesight (to eliminate the aphasic and memory component of the task). In addition, assistance was given if participants needed help to stabilize their performance or there was a threat of personal injury. Two trials were performed (separated within 45 min time window).

#### DF task

Patients were seated comfortably in front of a table. All of the objects were presented within reachable distance of the participant. An oral instruction was given to pierce the paper sheets and place them in the arch file. A picture showing paper put in the arch file was displayed to the patient, to avoid confounding factor of aphasia and memory. No time constraints were placed on the task performance.

### Apparatus

Sessions took place in designated laboratory space in the Hospital Bogenhausen Munich. Participants performance was recorded by a video camera located 45° to the right side of the table space. Fourteen objects were used in the TM task: electric kettle, teaspoon, two mugs, jug of water, container with tea bags, container with slices of lemon, jug of milk, jar of sugar cubes, sweetener dispense, saucer for used tea bags, dessert spoon, fork, and jar of coffee. The jar of coffee was used as a distractor item. Objects used in the DF task included two sheets of paper, a folder, a stapler, and a hole punch. The stapler was used as a distractor item.

### Data analysis

Each video recording was assessed by two researchers. Their scoring was averaged for each participant. Consistency of assessment reached 0.92 Kappa index (*P *<* *0.001) across individual ratings for the number of errors. For the analysis of the TM task an averaged performance was used between two trials and individual ratings. In DF, a time to complete the task was extracted from the video recording. The cut‐off scores for both task were determined with receiver operating characteristics (ROC) curve estimation. The BCoS subscales performance was assessed by a trained research neuropsychologist according to the scoring sheets provided with battery. For between group comparisons, Kruskal–Wallis test was used with Mann–Whitney test for post hoc comparisons. Spearman's rank correlational coefficient was used for correlational analysis between the neuropsychological scoring and ADL performance

## Results

### Clinical screening BCoS

**Figure 1:**
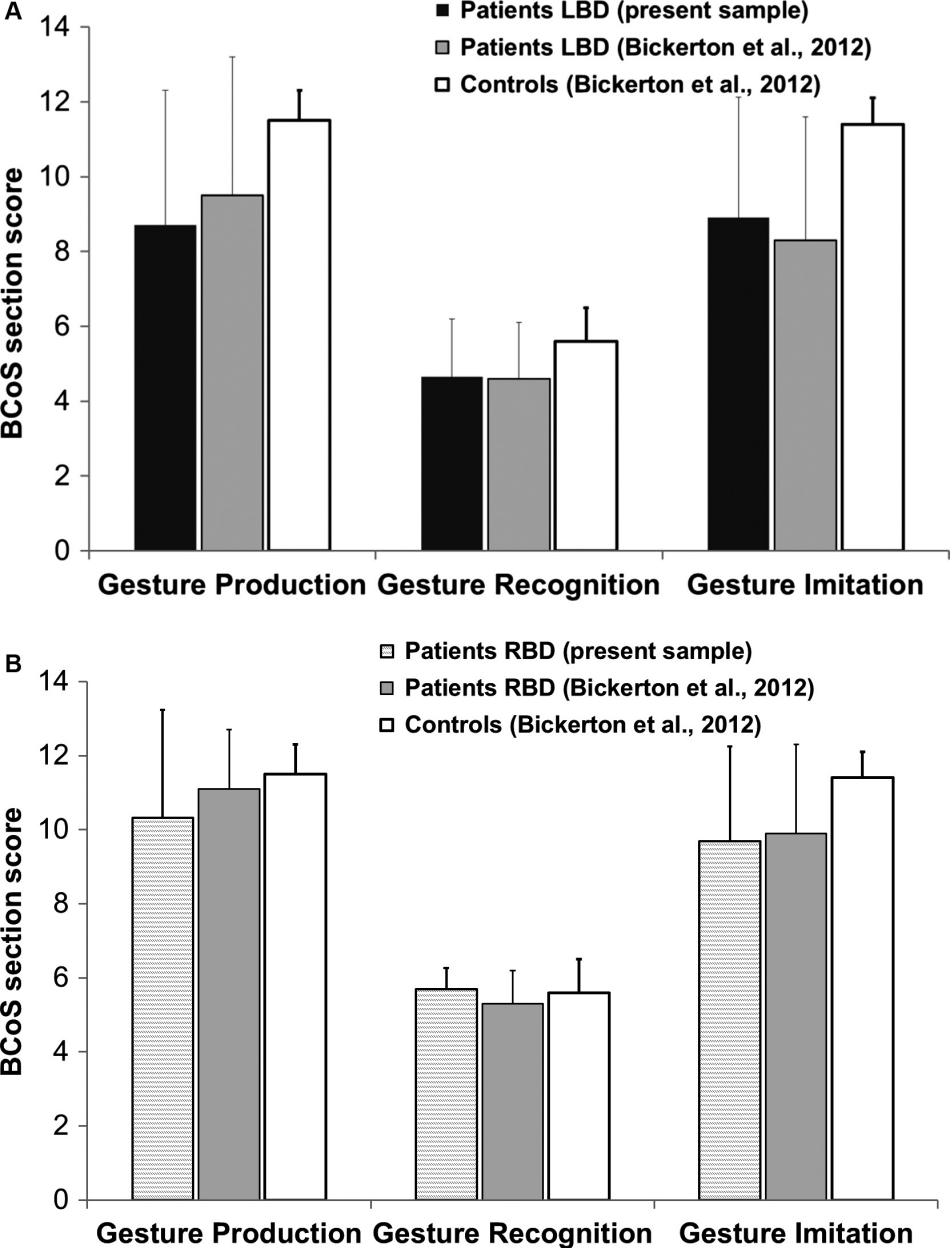
Illustration of the summary of scores on the praxis pantomime scales. LBD group top panel (A) and RBD group bottom panel (B). Scores of the LBD patients depicted were similar to the ones reported by Bickerton et al. (2012), (LBD *n* = 74; RBD *n* = 84). Likewise, RBD patients had similar scores to patients included in the Bickerton et al. (2012). Error bars denote standard deviation.

First we present the summary of results for the BCoS subscales and the two naturalistic tasks. Participant scored 1 point for correct performance of each item on the subscale, with a maximum of 12 points for gesture production and gesture imitation, and 5 points for gesture recognition. Figure 1 presents results for the LBD and RBD groups compared to the control and patient data published by Bickerton et al. (2012).

As summarized in the Table 3, both patient groups demonstrated mild‐to‐moderate neuropsychological deficits.

**Table 2:** Error taxonomy used to classify error types

Error type	Definitions	Example
Addition	Adding an extra component action that is not required in the action sequence	Adding instant coffee to cup 2
Anticipation	Performing an action earlier than usual	Turning the kettle on before pouring water into the kettle
Execution	An error in the execution of the task	Dropping the sweetener dispenser onto the table
Ingredient omission	Failing to add an ingredient required to complete the task goal	Failing to put sugar into cup 1
Misestimation	Using grossly too much or too little of some substance	Pouring half of the milk jug contents into cup 2
Mislocation	An action that is appropriate to the object in hand but is performed in completely the wrong place	Pouring some liquid from the bottle onto the table rather than into the glass
Ingredient substitution	An intended action carried out with an unintended ingredient	Pouring coffee grounds instead of sugar into cup 2
Perplexity	A delay or hesitation in performing an action	Picking up a tea bag and then pausing for an extended amount of time before placing it into a cup
Perseveration	The unintentional repetition of a step or subtask	Adding more than one tea bag to a cup
Object substitution	An intended action carried out with an unintended object	Pour heated water into non‐cup 1 object
Quality	The action was carried out, but not in an appropriate way	Putting the tea bag and the paper label into a cup
Sequence	Performing an action much later than usual	Switch kettle on after preparing both cups of tea
Sequence omission	An action sequence in which one step or subtask is not performed, despite the lack of any intention to omit the step or subtask	Turning on the kettle on without having inserted water

Error taxonomy was adapted from Hughes et al. 2014.

### TM task

**Figure 2:**
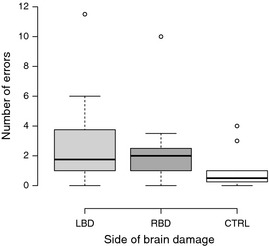
Average number of errors committed by participants during the tea making task. Error bars illustrate the range of errors.

**Figure 3:**
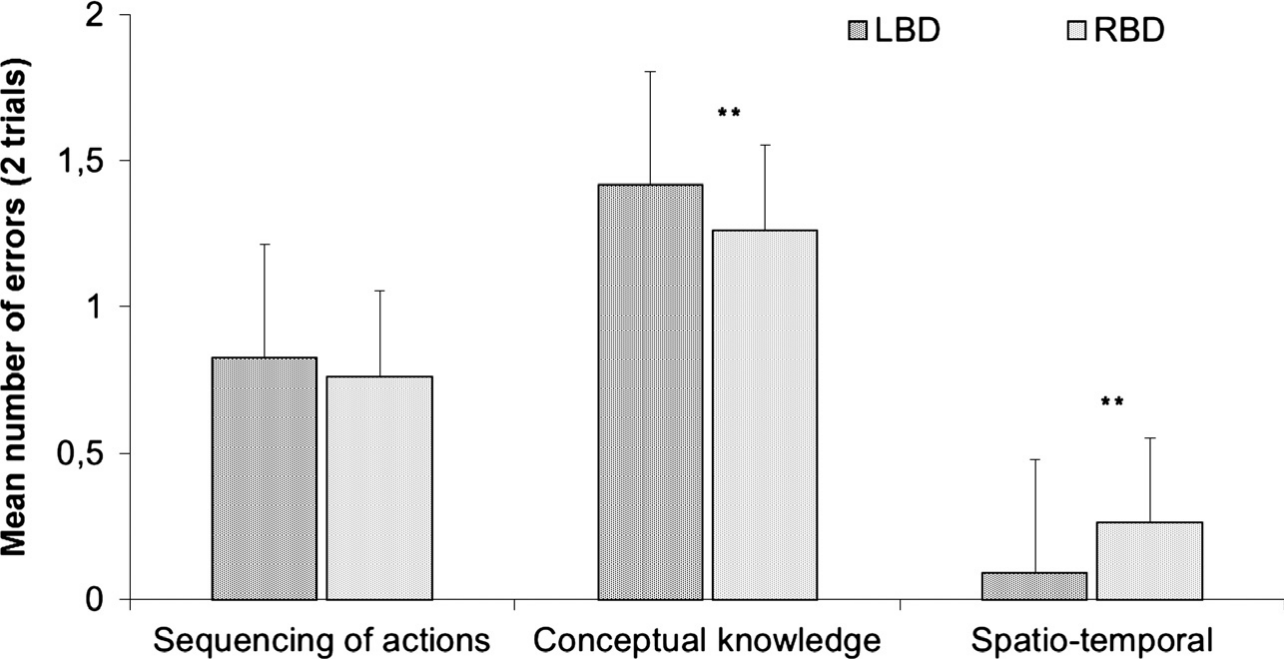
Summary of errors according to a novel classification in the tea making performance in patients. Error bars denote standard error. **Significant differences between error frequencies, *P* < 0.001.

Figure 2 depicts average number of all action errors committed during the trial by each participant group and Figure 3 illustrates the overview of errors committed by patients across two trials. Task performance in the TM was impaired for both LBD and RBD patients. On average, LBD participants committed 2.35 errors (SD = 2.26), while RBD participants committed 2.29 errors (SD = 2.18). In comparison, control group committed an average of 0.95 errors (SD = 1.19). Kruskal–Wallis test demonstrated differences between three groups for conceptual error category (χ^2^ [2, *N* =67] = 18.8, *P *<* *0.001) and spatiotemporal error category (χ^2^ [2, *N* =67] = 38.1, *P *<* *0.001), but not for sequencing errors. Comparison between LBD and RBD groups revealed significant differences in the number of errors committed in conceptual and spatiotemporal error categories (*z* = −3.6, *P *<* *0.0001; *z* = −5.4, *P *<* *0.001) (conceptual errors ranks LBD—33.1 and RBD—16.5; spatiotemporal ranks LBD—21.4 and RBD—42.39). On the basis of the total number of errors in the control and patient groups, we used ROC calculation with 0.95 confidence interval to determine cut‐off threshold for AADS behavior, with fair result (0.75 for TM, *P *<* *0.05; optimized for sensitivity and specificity with 1.25 error per trial as a cut‐off score of abnormal performance). In total, 55% of LBD and 55% of RBD sample committed more than 1.25 errors per trial in TM task.

**Table 3:** BCoS subscores

BCoS subtest	MOT		Apple test				Extinction				Complex Figure	
			Asymmetry complete		Asymmetry incomplete		Visual Extinct		Tactile Extinct			
Group/Value	*M*	SD	*M*	SD	*M*	SD	*M*	SD	*M*	SD	*M*	SD
LBD	11.5	1.2	0.2	1.2	0.1	0.9	0.0	1.2	−0.5	2.7	37.8	11.1
RBD	10.6	1.4	2.5	4.3	3.6	4.8	0.4	6.2	4.5	5.6	29.8	15.3
Norm scores (under 64 years of age)	11		¦<2¦		¦<2¦		¦<2¦		¦<2¦		42	

BCoS, Birmingham cognitive screen; *M*, average value for the group; SD, standard deviation for the group; ¦*n*¦, absolute value. Norm scores were taken from the Bickerton et al. 2012. In the spatial attention tests (apple and extinction) minus value denote left‐sided problems with attention; positive values indicate right‐sided deficits of attention.

In summary, both groups of patients showed impaired performance in the multistep tea preparation task. LBD patients had more pronounced problems with tool‐related knowledge in this task, manifesting as higher number of conceptual errors, such as object substitution or incorrect action for tool. RBD patients demonstrated more problems spatiotemporal aspects of movement performance. These deficits manifested as inadequate spatial positioning and handling of the objects, inefficient grasp, or problems with fine motor control.

### Document filing

**Figure 4:**
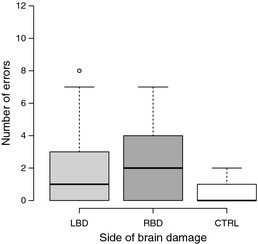
Average number of errors committed by participants during the document filing task.

**Figure 5:**
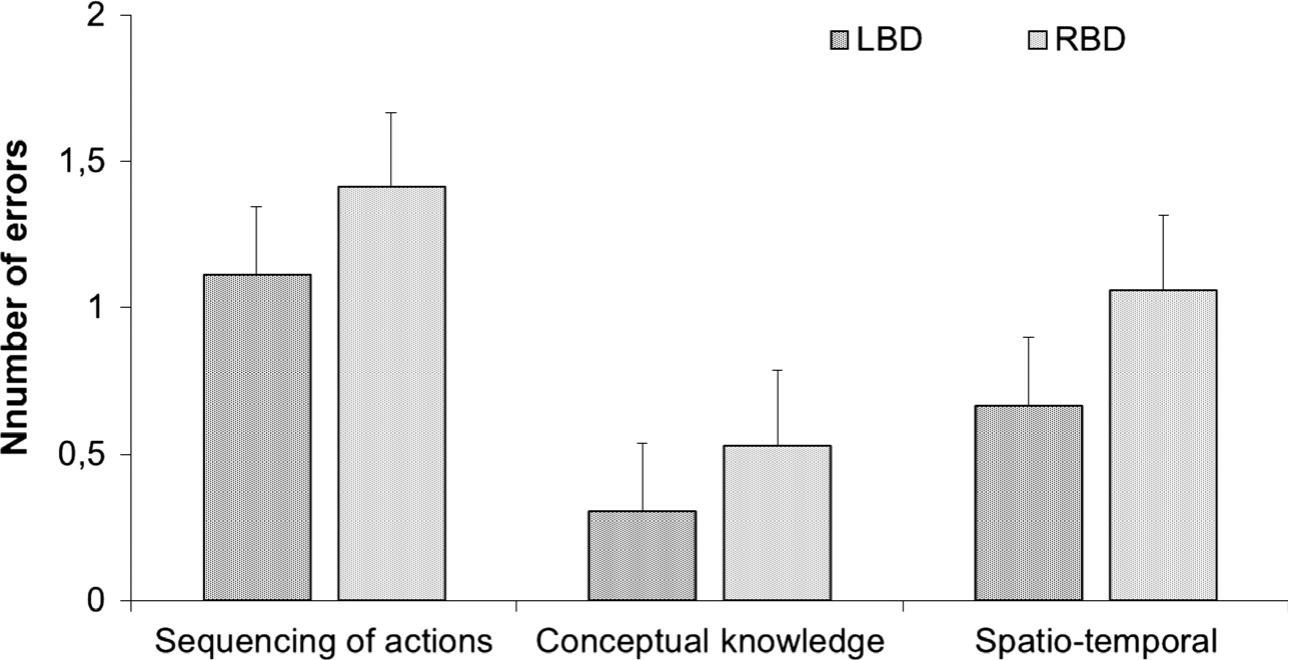
Depiction of global error categories applied to the document filing averaged across participants. Error bars denote standard error.

Figure 4 depicts the average number of all errors committed during the trial by each participant. In the DF task, participants with LBD and RBD showed impaired task performance. On average, LBD patients committed 1.94 errors (SD = 2.24) and RBD patients committed 2.29 (SD = 2.59). The control group committed an average of 1.18 errors in the sequence, 0.29 in the conceptual, and none in the spatiotemporal category. Figure 5 presents overview of errors committed by patient groups. Kruskal–Wallis test demonstrated differences between three groups for sequence error category (χ^2^ [2, *N* = 67] = 13.6 *P *=* *0.001) and spatiotemporal error category (χ^2^ [2, *N* = 67] = 6.8, *P *<* *0.05), but not for conceptual errors. Comparison between LBD and RBD groups does not reveal significant differences between error frequencies in the three error categories. Using ROC curve estimation we set a cut‐off score of 1.25 error per trial as abnormal criteria (test result 0.70 for DF and *P *<* *0.05; optimized for sensitivity and specificity). In total, 37% of LBD and 50% RBD presented erratic performance in DF task.

### Neuropsychological correlates of deficits in AADS

The next section presents findings from the correlation analysis between the TM and DF performance and the BCoS scores along with AAT. For each patient group different subtests of BCoS were taken into consideration based on the descriptive statistics presented in the previous section. For example, apple test scores were not taken into consideration for the LBD group because with the exception of mild attentional deficits this patient group does not suffer from compromised spatial attention (e.g., neglect). Therefore, based on the differences in the BCoS scores, the RBD and LBD patients were separated for the purpose of correlation analysis. The presentation of results is divided into two parts. First, we included the global categories of the action errors observed in patients during both tasks. Second, we looked into links between particular errors (prevalent in the RBD group).

#### LBD patients

Correlation analysis revealed no significant relationship between total number of action errors and errors classified in global categories in the TM and DF tasks, suggesting that the tasks involved different sets of cognitive resources. In the LBD group, analysis was conducted with the inclusion of AAT scores. Summary of correlation matrix is presented in Table 4. No relationship was found between the AAT scores and global error categories (*P *>* *0.05). However, we found strong correlations between TT PR and praxis scales with gesture performance and gesture recognition (TT PR—gesture production: *r* = 0.5, *P *<* *0.01 and TT PR—gesture recognition: *r* = 0.43, *P *<* *0.05); and negative correlation between written language and naming with gesture recognition (written language—gesture recognition: *r* = −0.39, *P *=* *0.04 and naming—gesture recognition: *r* = −0.45, *P *=* *0.01). Praxis subscales correlated strongly with performance in both tasks. Gesture production, gesture recognition, and gesture imitation were significantly negatively correlated with the deficits in sequencing the TM task. In the DF task, gesture recognition and gesture imitation were significantly negatively correlated with the number of committed conceptual errors. Moreover, we found that tactile extinction (deficit in spatial attention on the contralesional side) deficits were significantly associated with sequencing difficulties in the DF task.

**Table 4:** Summary of correlations between neuropsychological assessment scores and performance in TM and DF tasks

		Tea making				Document filing			
		Sequence	Concept	Spatiotemporal	All errors	Sequence	Concept	Spatiotemporal	All errors
*LBD*									
Neglect	Space/Obj.	0.2/0.02	0.22/0.05	0.32/−0.05	0.1/0.03	0.31/0.03	0.30/0.20	0.1/−0.14	0.07/0.10
Extinction	Visual/Tact.	0.1/0.03	−0.09/0.16	−0.23/0.1	0.08/0.09	0.05/**0.35** *	0.19/0.06^mis^ *	−0.13^exe^ */0.24	0.07/0.25
Apraxia	Panto	−**0.42** **	−0.07	0.12	−0.29	−0.07	−0.25	−0.01	−0.18
	Imit	−**0.43** **	−0.06	0.14	−**0.36** *	0.02	−**0.38** *	−0.09	−0.14
Figure Copy		−0.19	−0.25	−0.2	−0.21	−0.07	−0.06	−0.05	−0.18
Aphasia	TT	−0.24	−0.06	−0.13	−0.05	−0.12	−0.21	0.06	−0.3
	Naming	−0.09	−0.13^mis^ *	0.09	0.1	0.24	0.38^t^	0.17	0.3
	Compreh.	−0.27	−0.29^mis^ *	−0.08	0.01	0.13	0.31	0.24	0.29
*RBD*									
Neglect	Space/Obj.	−21	0.09/0.06	0.14/−0.06	0.17/0.08	0.02/−0.21	−0.06/−0.21^mis^ *	0.29/0.46	0.38/0.09
Extinction	Visual/Tact.	0.38/−0.18	−0.19^ml^ */0.01	−**0.49** */0.22	−0.16/0.15	−0.3/0.32	−0.36^ml^ */0.02	−0.23/0.23	−0.33/0.28
Apraxia	Panto	−**0.56** *	−0.36	−0.11	−0.35	−0.36	−**0.55** *	−0.42^t^	−**0.54** *
	Imit	−0.41	−0.29	−**0.66** **	−0.26	−**0.53** *	−0.31	−0.22	−0.28
Figure Copy		−0.15	−0.27^ml^ *	−0.46^t^	−**0.57** *	−**0.57** *	−0.33	−**0.57** *	−**0.54** *

Shaded gray areas in the right panel denote screening scores below the norm for the patient sample. Bold values in the correlation matrix denote significant correlation of screening scores with global error category. *P < 0.05, **P<0.01, *t*−trending significance value, *P* < 0.1, significant correlations with error types: ml, mislocation; mis, misestimation; exe, execution; LBD, left brain damage; RBD, right brain damage.

In addition, we evaluated correlations between error types (within global categories of errors) with the neuropsychological assessment. In TM task, we found a correlation between the number of misestimation errors and scores on the tactile extinction scale of BCoS (*r* = 0.43, *P *<* *0.01), and execution errors and scores on the visual extinction scale of BCoS (*r* = −0.38, *P *=* *0.01). In addition, we found a negative correlation of misestimation error with AAT subtests (repetition, *r* = −0.44, *P *<* *0.05; naming, *r* = −0.39, *P *<* *0.05; comprehension, *r* = −0.47, *P* < 0.05) and correlation trend with written subtest (*r* = −0.35, *P* = 0.68). In the DF task, the number of misestimation errors correlated negatively with gesture recognition (*r* = −0.36, *P* < 0.05) and gesture imitation scores were negatively associated with quality errors in this task (*r* = −0.35, *P* < 0.05). Gesture imitation also correlated with complex figure copy performance (*r* = 0.47, *P* < 0.01). No main effect was found for motor impairment on task performance (total number of errors) and other clinical measures taken from the neuropsychological assessment.

#### RBD patients

In RBD patients, visual attention is often compromised by left‐sided neglect. Therefore, we expected that the presence of neglect and visual extinction might influence performance in examined tasks. Summary of correlation matrix is presented in Table 4. We found significant relationship between egocentric apple test score (indicative of neglect in the BCoS) and the MOT subtest of the BCoS (*r* = 0.58, *P* < 0.01). However, there was no correlation between other global categories of errors and the apple test scores. Compromised visual extinction was found to negatively correlate with global error categories on spatiotemporal dimensions in the TM task. Interestingly, no relationship was found between tactile extinction and task performance despite task performance was clearly impaired in the RBD patients (Table 3). However, tactile extinction score was significantly correlated with egocentric and allocentric apple test score (*r* = 0.73, *P* < 0.001; *r* = 0.48, *P* = 0.05) and complex figure copy performance (*r* = 0.66, *P* < 0.01). Significant correlations were detected between different action errors in both tasks and various measures of apraxia. However, the above correlations with apraxia scores did not hold when partially controlled for performance in the spatial attention tests (*P* > 0.05), which suggests a strong contribution of spatial deficits to the difficulties with multistep actions in AADS and transitive and intransitive gestures. Likewise, the link between the sequencing errors in the DF task and the gesture imitation score did not reach significance when partially controlled for spatial attention tasks (*P* > 0.05). Finally, complex figure copy performance was linked with all the ADL tasks involved MOT, TM task, and DF task. This test involves many different cognitive functions that also contribute to the successful ADL functioning. Strong negative correlations were found—lower complex figure copy scores were associated with a higher frequency of errors in all ADL‐related tasks in RBD patients. In summary, the analysis of the correlates of impaired ADL performance in RBD revealed multifaceted links primarily with spatial attention.

In addition, we ran correlational analysis between error types (within the global error categories) and the neuropsychological data. Interestingly, mislocation errors in TM and DF were negatively correlated with visual extinction subscale scores (*r* = 0.60, *P* = 0.01; *r* = 0.54, *P* < 0.01). Mislocation errors between the tasks showed trend toward correlation (*r* = 0.46, *P* = 0.06). In TM task, mislocation errors were also correlated with praxis scales gesture recognition and gesture imitation scores (*r* = −0.57, *r* = −0.51, *P* < 0.05), showed a trend toward significance with gesture production (*r* = −0.44, *P* = 0.80) and correlated with complex figure copy scores (*r* = −0.51, *P* < 0.05). In addition, allocentric apple test score correlated with number of misestimation errors (*r* = 0.55, *P* < 0.05). In the DF task, we found negative correlations between misestimation errors and performance on the BCoS praxis subscale, gesture production (*r* = −0.64, *P* < 0.01). There was no main effect of paresis on task performance (total number of errors) and BCoS assessment.

## Discussion

### Summary of the findings

Similar to previous reports (e.g., Hartmann et al. 2005; Rexroth et al. 2005; Rumiati et al. 2005; Buxbaum 1998) we observed mild to severe impairments of both LBD and RBD patients, demonstrated as deficits in the tested ADL tasks (TM task and DF task). Those findings reinforce the stance that apraxia and action disorganization syndrome can be difficult to disentangle behaviorally, and that deficits in ADL performance are present in a substantial number of patients with unilateral lesions in either hemisphere (Humphreys and Forde 1998). In the TM task, we reported higher ratio of conceptual errors in LBD sample than RBD sample and higher ratio of the spatiotemporal errors in the RBD than in the LBD sample. In the DF task, the differences in descriptive statistics did not reach statistical significance. The results from TM are congruent with previous reports suggesting problems with conceptual knowledge in LBD patients and deficits in smooth motor control in the ipsilesional hand following stroke (see Goldenberg 2013, Chapter 13, for review). Both groups demonstrated difficulty with sequencing of action substeps in both tasks. Interestingly, performance on both ADL tasks was not correlated, suggesting that they involved a different set of higher cognitive functions. However, both tasks involved multiobject use and could be classified as multiple step actions.

Descriptive, comparative, and correlational analysis of the current dataset provided new insights into the discussion about comorbidity links between AADS and neuropsychological deficits following stroke. Results confirmed that apraxia poses critical limitations on ADL performance. In the LBD group, praxis pantomime scales were negatively correlated with action errors (sequencing and conceptual) in both tasks. However, the moderate to strong correlations indicate that apraxia alone cannot explain action impairments in those patients. We found that LBD group's deficits in tactile extinction were linked to difficulties with action sequencing and misestimation errors. Deficits in visual extinction correlated with execution errors in DF task. We reported no relationship between AAT scores and task performance (despite a correlation between AAT and pantomime praxis scales of BCoS) apart from one type of error, namely misestimation errors in TM. In comparison, Hartmann et al. (2005) reported correlations between token test score and performance in a drip‐maker coffee preparation as well as pantomime assessment, but not in the use of cassette recorder. Thus, our expectation to find a relationship in LBD patients between aphasia assessment and global error categories for ADL task performance was not confirmed.

Analysis of the RBD sample revealed a strong correlation between compromised spatial attention (due to hemineglect, visual extinction, or hemianopia) and spatial aspects of task performance in both tasks, namely mislocation and misestimation errors. All together we confirmed our hypothesis that compromised spatial attention might contribute to AADS difficulties in the RBD sample, and project onto ADL functioning of patients. Interestingly, unlike in the LBD group the link between tactile extinction and task performance was nonsignificant for RBD patients. In addition, complex figure copy performance in RBD patients correlated negatively with performance in TM, DF, and MOT. This unexpected finding suggests that the use of complex figure copy in clinical assessment might be an indirect measure of deficits in ADL functioning in RBD patients.

### Comparison of findings of this study with the previous research

The results of this study partially support previous reports investigating LBD and RBD performance in the context of daily independence.

#### Overall error production of LBD and RBD

In sum, a plethora of research demonstrated that RBD patients show preserved performance of single tool actions, but are impaired to a similar extent with LBD patients in the multistep actions. Similarly to the previously mentioned authors, we argue that although the behavioral manifestations of AADS are difficult to disentangle between the unilateral damage groups, they might have different functional origin (Schwartz et al. 1999; Hartmann et al. 2005; Rumiati et al. 2005, Poole et al. 2011). It was hypothesized that decreased independence in RBD patients relates to the depletion of working memory or resources capacity, whereas the difficulties in LBD refer more to compromised cognitive knowledge (Schwartz et al. 1999; Rumiati et al. 2005; Goldenberg 2013). In a study by Goldenberg et al. (2007), patients with frontal lesions and diagnosed dysexecutive syndrome also suffered from impaired ability to perform multistep actions. In sum, previous research supports our findings that RBD patients are equally impaired on ADL type of activities as LBD patients, but the origin of impairment might be different in both groups (Buxbaum 1998).

#### Gestural praxis and naturalistic action production

First, we reported links between the praxis scales and the ADL performance (number of errors) in both groups of patients. Therefore, in our sample of LBD patients we assume that the low scores on gestural praxis‐related tasks coincided with poor ADL performance. In RBD group, those correlations lost significance when spatial attention tasks were partially controlled for (tactile and visual extinction). The relationship between gestural praxis and actual tool use is complex with mixed relationship in the literature. Consistent with current report, Clark et al. (1994) demonstrated that LBD patients show impairment in planning the movement of the hand both for the pantomime performance and actual tool use. The authors used a bread slicing task in naturalistic scenario with and without actual tool execution. Likewise, Buxbaum and Saffran (2002) found that impaired gestural praxis in LBD patients coincides with impaired tool and manipulation knowledge and these relationships are independent from the overall cognitive impairment caused by stroke. Furthermore, Goldenberg and Hagmann (1998) reported that impairment with familiar tool use is indeed linked to deficits in gestural praxis, but only if LBD patients also show deficits in mechanical problem solving. This point was also raised by Hermsdörfer et al. (2006) suggesting that discrepancy of errors and kinematic profile between pantomime and actual execution might be an indication of independent deficits. Likewise, Laimgruber et al. (2005) proposed that actual object manipulation execution imposes mechanical constraints and affordances absent in pantomime and the translation of deficits from one performance to another is not directional. This stance is supported by clinical reports of cases where patients are impaired on one task, but not on the other (Rumiati et al., 2001). Therefore, in our sample of LBD patients we assume that the low scores on gestural praxis‐related tasks coincided with poor ADL performance, but might not have the same functional substrate as originally posited by Liepmann (1905).

#### Aphasia and naturalistic action production

Second, the relationship between severity of aphasia and impaired ADL performance remains unclear. In our study, we found no correlation between language capacity and ADL task performance, apart from one error type—misestimation. The assumption that language and movement functions are anatomically linked together comes from the early investigations of apraxia conducted by Hugo Liepmann (1905), one of the pioneers of apraxia research. Originally, he proposed that apraxia was caused by impairment in “movement formulae”; an action depiction composed of visuoacoustic elements. In his study, he reported an increased rate of apraxia in aphasic individuals with right hemiplegia in comparison to nonaphasic individuals (Goldenberg 2013; Chapter 2). Liepmann proposed that left hemisphere is critical not only for language but also for movement. Importantly, he posited that apraxia is not caused by aphasia, but is likely to coincide due to similar neural underpinning in the brain. In the seminal report by Hartmann et al. (2005), severity of aphasia (measured with AAT) in LBD was negatively correlated to performance in the coffee‐making task. With more severe language impairment, less independence was observed during this task. However, this relationship was not observed in the second task included in the study, namely cassette recorder use. This task was linked by the authors to multistep mechanical object use. Importantly, the LBD sample consisted exclusively of aphasic individuals. In our study only 80% of patients in LBD sample were diagnosed with aphasia. We acknowledge that the difference between these two reports might be caused by different inclusion criteria in the tested population. It is important to note that other studies, such as a recent report by Poole et al. (2011) on meal preparation behavior, found no relationship between aphasia severity and number of errors in the LBD sample. This is consistent with the reports from Rumiati et al. (2001) and Buxbaum et al. (1998) who demonstrated that even with preserved semantic lexical knowledge of tool use, patients with LBD can show dramatic impairments in naturalistic action production.

#### Spatial attention and naturalistic action production

Finally, in the previous sections we proposed that compromised spatial attention can manifest in compromised gestural praxis performance impairment in RBD. Similar to Buxbaum (1998), Hartmann et al. (2005), and Poole et al. (2011) we found a significant link between the spatial attention functions and the naturalistic performance not only in RBD patients, but also in LBD. Likewise, we did not qualitatively observe during video assessment a relationship between spatial location of errors made and properties of the workspace. We found adverse effect of visual extinction manifests in increased rate of mislocation errors (in both LBD and RBD groups) and allocentric neglect indication correlated with misestimation error in RBD group. In addition, other research groups reported that visuospatial neglect is a robust predictor for the functional outcome of ADL independence in the posthospitalization period (Denes et al. 1982; Edmans and Lincoln, 1991; Katz et al. 1999;  Jehkonen et al. 2000; Paolucci et al., 1998). To the best of our knowledge, this is the first study that emphasizes the deficits in extinction as detrimental for ADL independence in stroke patients.

These findings are important for understanding the different origins of AADS impairments and consequently the choice of rehabilitation strategy aimed at increasing daily independence of stroke patients. In the report of Walker et al. (2012) on their intervention trial targeted at improving dressing independence, two different approaches were compared across LBD and RBD groups. The first intervention was based on neuropsychological approach targeted at ameliorating the spatial attention difficulties, while the second intervention was based on a standard occupational therapy approach. RBD patients benefited more (measured as increased dressing independence) with neuropsychological training tailored at spatial attention deficits, whereas there was no differences in LBD group in terms of efficacy of both interventions. This suggests that in the LBD group both occupational and neuropsychological therapy brings similar improvement in function. Other more generic approaches to broadening spatial attention were proposed by Làdavas (2008) and Kalra et al. (1997) in the context of improving overall functioning in stroke survivors. In sum, these approaches support the notion that standard occupational therapy should be assisted with neuropsychological training focused on spatial attention impairments. This study confirmed that presence of those impairments affects the ADL independence in stroke survivors.

#### Limitations

Although we found a satisfactory number of patients for this study, the results demonstrated are not giving a clean cut answer to our main research question. Is there a relationship between deficits in neuropsychological functions and ADL performance? We found substantial evidence that in both LBD and RBD patients there is a complex relationship between gestural praxis ability, spatial attention, and ADL performance. One of the limitations of this study was the fact that patients were tested within a broad time frame since the stroke, inclusive of subacute and chronic patients. Other limitations related to a lack of tests for mechanical problem solving and semantic memory of the tool use included in the testing session. This was due to the limited time allocated for the experimental session within the hospital.

## Conclusions

In sum, analysis of neuropsychological correlates of AADS in LBD and RBD patients revealed that both groups might suffer from compromised ADL independence. That said, despite similar behavior, the underlying mechanisms of those deficits might be different. In our sample, we found that praxis performance and compromised spatial attention (extinction and neglect) are linked to poor performance in both groups of patients. Taking into consideration previous work by Poole et al. (2011), Schwartz et al. (1999), and Hartmann et al. (2005), we pose a question whether AADS can be completely disambiguated from the impact of comorbidity syndromes on ADL independence.

## Conflict of Interest

None declared.

## References

[brb3371-bib-0001] Bickerton, W.‐L. , G. W. Humphreys , and M. J. Riddoch . 2007 The case of the unfamiliar implement: schema‐based over‐riding of semantic knowledge from objects in everyday action. J. Int. Neuropsychol. Soc. 13:1035–1046. 17942021 10.1017/S1355617707071585

[brb3371-bib-0002] Bickerton, W.‐L. , D. Samson , J. Williamson , and G. W. Humphreys . 2011 Separating forms of neglect using the Apples Test: validation and functional prediction in chronic and acute stroke. Neuropsychology 25:567–580. 21574718 10.1037/a0023501

[brb3371-bib-0003] Bickerton, W.‐L. , M. J. Riddoch , D. Samson , A. B. Balani , B. Mistry , and G. W. Humphreys . 2012 Systematic assessment of apraxia and functional predictions from the Birmingham Cognitive Screen. J. Neurol. Neurosurg. Psychiatry 83:513–521. 22383734 10.1136/jnnp-2011-300968

[brb3371-bib-0004] Bienkiewicz, M. M. N. , M.‐L. Brandi , G. Goldenberg , C. M. L. Hughes , and J. Hermsdörfer . 2014 The tool in the brain: apraxia in ADL. Behavioral and neurological correlates of apraxia in daily living. Front. Psychol. 5, 353. 24795685 10.3389/fpsyg.2014.00353 PMC4005934

[brb3371-bib-0005] Buxbaum, L. J. 1998 Ideational apraxia and naturalistic action. Cogn. Neuropsychol. 15:617–643. 22448839 10.1080/026432998381032

[brb3371-bib-0105] Buxbaum, L. J. , M. F. Schwartz , and T. G. Carew . 1997 The role of semantic memory in object use. Cogn. Neuropsychol. 14:219–254.

[brb3371-bib-0006] Buxbaum, L. J. , T. Giovannetti , and D. Libon . 2000 The role of the dynamic body schema in praxis: evidence from primary progressive apraxia. Brain Cogn. 44, 166–191. 11041988 10.1006/brcg.2000.1227

[brb3371-bib-0101] Buxbaum, L. J. , and E. M. Saffran . 2002 Knowledge of object manipulation and object function: dissociations in apraxic and nonapraxic subjects. Brain Lang. 82:179–199. 12096875 10.1016/s0093-934x(02)00014-7

[brb3371-bib-0007] Buxbaum, L. J. , A. Shapiro , and H. B. Coslett . 2014 Critical brain regions for tool related and imitative actions: a componential analysis. Brain 137:1971–1985. 24776969 10.1093/brain/awu111 PMC4065019

[brb3371-bib-0008] Chechlacz, M. , P. Rotshtein , and G. W. Humphreys . 2012 Neuroanatomical dissections of unilateral visual neglect symptoms: ALE meta‐analysis of lesion‐symptom mapping. Front. Human Neurosci. 6:230. doi:10.3389/fnhum.2012.00230. 10.3389/fnhum.2012.00230 PMC3415822 22907997

[brb3371-bib-0009] Chechlacz, M. , A. Terry , N. Demeyere , H. Douis , W.‐L. Bickerton , P. Rotshtein , et al. 2013 Common and distinct neural mechanisms of visual and tactile extinction: a large scale VBM study in sub‐acute stroke. Neuroimage Clin. 2, 291–302. 24179784 10.1016/j.nicl.2013.01.013 PMC3777674

[brb3371-bib-0010] Chechlacz, M. , A. Novick , P. Rotshtein , W.‐L. Bickerton , G. W. Humphreys , and N. Demeyere . 2014 The neural substrates of drawing: a voxel‐based morphometry analysis of constructional, hierarchical, and spatial representation deficits. J. Cogn. Neurosci. 26:1–15. 24893744 10.1162/jocn_a_00664

[brb3371-bib-0102] Clark, M. A. , A. S. Merians , A. Kothari , H. Poizner , B. Macauley , L. J. Gonzalez Rothi , et al. 1994 Spatial planning deficits in limb apraxia. Brain 117(Pt 5):1093–1106. 7953591 10.1093/brain/117.5.1093

[brb3371-bib-0011] Denes, G. , C. Semenza , E. Stoppa , and A. Lis . 1982 Unilateral spatial neglect and recovery from hemiplegia. Brain 105, 543–552. 7104665 10.1093/brain/105.3.543

[brb3371-bib-0012] Driver, J. , and P. Vuilleumier . 2001 Perceptual awareness and its loss in unilateral neglect and extinction. Cognition 79, 39–88. 11164023 10.1016/s0010-0277(00)00124-4

[brb3371-bib-0103] Edmans, J. A. , and N. B. Lincoln . 1991 Treatment of visual perceptual deficits after stroke: single case studies on four patients with right hemiplegia. Br. J. Occup. Ther. 54:139–144.

[brb3371-bib-0104] Goldenberg, G. , J. Hermsdörfer , R. Glindemann , C. Rorden , and H.‐O. Karnath . 2007 Pantomime of tool use depends on integrity of left inferior frontal cortex. Cereb. Cortex 17:2769–2776. 17339607 10.1093/cercor/bhm004

[brb3371-bib-0014] Goldenberg, G . 2013 Apraxia: The cognitive side of motor control. Oxford Univ. Press, Oxford, U.K. 10.1016/j.cortex.2013.07.016 24144066

[brb3371-bib-0106] Goldenberg, G. , and S. Hagmann . 1998 Tool use and mechanical problem solving in apraxia. Neuropsychologia 36:581–589. 9723930 10.1016/s0028-3932(97)00165-6

[brb3371-bib-0015] Hanna‐Pladdy, B. , K. M. Heilman , and A. L. Foundas . 2003 Ecological implications of ideomotor apraxia: evidence from physical activities of daily living. Neurology 60:487–490. 12578932 10.1212/wnl.60.3.487

[brb3371-bib-0016] Hartmann, K. , G. Goldenberg , M. Daumüller , and J. Hermsdörfer . 2005 It takes the whole brain to make a cup of coffee: the neuropsychology of naturalistic actions involving technical devices. Neuropsychologia 43:625–637. 15716152 10.1016/j.neuropsychologia.2004.07.015

[brb3371-bib-0107] Hermsdörfer, J. , S. Hentze , and G. Goldenberg . 2006 Spatial and kinematic features of apraxic movement depend on the mode of execution. Neuropsychologia 44:1642–1652. 16678222 10.1016/j.neuropsychologia.2006.03.023

[brb3371-bib-0108] Hermsdörfer, J. , Y. Li , J. Randerath , G. Goldenberg , and L. Johannsen . 2012 Tool use without a tool: kinematic characteristics of pantomiming as compared to actual use and the effect of brain damage. Exp. Brain Res. 218:201–214. 22349499 10.1007/s00221-012-3021-z

[brb3371-bib-0109] Hoeren, M. , D. Kümmerer , T. Bormann , L. Beume , V. M. Ludwig , M.‐S. Vry , et al. 2014 Neural bases of imitation and pantomime in acute stroke patients: distinct streams for praxis. Brain 137(Pt 10):2796–2810. 25062694 10.1093/brain/awu203

[brb3371-bib-0017] Huber, W. , K. Poeck , and K. Willmes . 1984 The Aachen Aphasia Test. Adv. Neurol. 42, 291–303. 6209953

[brb3371-bib-0018] Hughes, C. M. , C. Baber , M. M. N. Bienkiewicz , A. Worthington , A. Hazell , and J. Hermsdörfer . 2014 The application of SHERPA (Systematic Human Error Reduction and Prediction Approach) in the development of compensatory cognitive rehabilitation strategies for stroke patients with left and right brain damage. Ergonomics 15:1–21. 10.1080/00140139.2014.957735 25222822

[brb3371-bib-0019] Humphreys, G. W. , and E. M. E. Forde . 1998 Disordered action schema and action disorganisation syndrome. Cogn. Neuropsychol. 15:771–811.

[brb3371-bib-0020] Humphreys, G. W. , W.‐L. Bickerton , D. Samson , and M. Riddoch . 2012 Birmingham cognitive screen (BCoS). Psychology Press, Hove. 10.1136/jnnp-2011-300968 22383734

[brb3371-bib-0021] Jehkonen, M. , J.‐P. Ahonen , P. Dastidar , A.‐M. Koivisto , P. Laippala , J. Vilkki , et al. 2000 Visual neglect as a predictor of functional outcome one year after stroke. Acta Neurol. Scand. 101:195–201. doi:10.1034/j.1600‐0404.2000.101003195.x. 10705943 10.1034/j.1600-0404.2000.101003195.x

[brb3371-bib-0022] Jehkonen, M. , M. Laihosalo , and J. E. Kettunen . 2006 Impact of neglect on functional outcome after stroke: a review of methodological issues and recent research findings. Rest. Neurol. Neurosci. 24:209–215. 17119299

[brb3371-bib-0114] Kalra, L. , I. Perez , S. Gupta , and M. Wittink . 1997 The influence of visual neglect on stroke rehabilitation. Stroke 28:1386–1391. 9227688 10.1161/01.str.28.7.1386

[brb3371-bib-0023] Katz, N. , A. Hartman‐Maeir , H. Ring , and N. Soroker . 1999 Functional disability and rehabilitation outcome in right hemisphere damaged patients with and without unilateral spatial neglect. Arch. Phys. Med. Rehabil. 80, 379–384. 10206598 10.1016/s0003-9993(99)90273-3

[brb3371-bib-0115] Làdavas, E. 2008 Multisensory‐based Approach to the recovery of unisensory deficit. Ann. N. Y. Acad. Sci. 1124:98–110. 18400926 10.1196/annals.1440.008

[brb3371-bib-0116] Laimgruber, K. , G. Goldenberg , and J. Hermsdörfer . 2005 Manual and hemispheric asymmetries in the execution of actual and pantomimed prehension. Neuropsychologia 43:682–692. 15721181 10.1016/j.neuropsychologia.2004.09.004

[brb3371-bib-0117] Liepmann, H. 1905 Der weitere Krankheitsverlauf bei dem einseitig Apraktischen und der Gehirnbefund auf Grund von Serienschnitten. Eur. Neurol. 17:289–311.

[brb3371-bib-0024] Meyers, J. E. , and K. R. Meyers . 1995 Rey complex figure copy test and recognition trial: professional manual. Psychological Assessment Resources, Odessa, FL.

[brb3371-bib-0110] Paolucci, S. , G. Antonucci , and M. Grazia . 1998 Functional Outcome in Stroke Inpatient Rehabilitation: Predicting No, Low and High, 228–234. 10.1159/000015856 9684063

[brb3371-bib-0025] Poole, J. L. , J. Sadek , and K. Y. Haaland . 2011 Meal preparation abilities after left or right hemisphere stroke. Arch. Phys. Med. Rehabil. 92:590–596. doi:10.1016/j.apmr.2010.11.021. 21440704 10.1016/j.apmr.2010.11.021

[brb3371-bib-0111] Rexroth, P. , A. G. Fisher , B. K. Merritt , and J. Gliner . 2005 ADL differences in individuals with unilateral hemispheric stroke. Can. J. Occup. Ther. 72:212–221. 16252608 10.1177/000841740507200403

[brb3371-bib-0026] Rothi, L. J. , and K. M. Heilman . 1997 Apraxia: The neuropsychology of action. Psychology Press, Hove.

[brb3371-bib-0112] Rumiati, R. I. , S. Zanini , L. Vorano , and T. Shallice . 2001 A form of ideational apraxia as a delective deficit of contention scheduling. Cogn. Neuropsychol. 18:617–642. 20945230 10.1080/02643290126375

[brb3371-bib-0113] Rumiati, R. I. , P. H. Weiss , A. Tessari , A. Assmus , K. Zilles , H. Herzog , et al. 2005 Common and differential neural mechanisms supporting imitation of meaningful and meaningless actions. J. Cogn. Neurosci. 17:1420–1431. 16197695 10.1162/0898929054985374

[brb3371-bib-0027] Schwartz, M. F. , L. J. Buxbaum , M. W. Montgomery , E. Fitzpatrick‐DeSalme , T. Hart , M. Ferraro , et al. 1999 Naturalistic action production following right hemisphere stroke. Neuropsychologia 37:51–66. 9920471 10.1016/s0028-3932(98)00066-9

[brb3371-bib-0028] Sunderland, A. , C. M. Walker , and M. F. Walker . 2006 Action errors and dressing disability after stroke: an ecological approach to neuropsychological assessment and intervention. Neuropsychol. Rehabil. 16:666–683. 17127572 10.1080/09602010500204385

[brb3371-bib-0029] Wade, D. T. , and R. L. Hewer . 1987 Functional abilities after stroke: measurement, natural history and prognosis. J. Neurol. Neurosurg. Psychiatry 50, 177–182. 3572432 10.1136/jnnp.50.2.177 PMC1031489

[brb3371-bib-0030] Walker, M. F. , A. Sunderland , J. Fletcher‐Smith , A. Drummond , P. Logan , J. A. Edmans , et al. 2012 The DRESS trial: a feasibility randomized controlled trial of a neuropsychological approach to dressing therapy for stroke inpatients. Clin. Rehabil. 26:675–685. 22180445 10.1177/0269215511431089 PMC3479683

